# Human fetal inner ear involvement in congenital cytomegalovirus infection

**DOI:** 10.1186/2051-5960-1-63

**Published:** 2013-10-02

**Authors:** Liliana Gabrielli, Maria Paola Bonasoni, Donatella Santini, Giulia Piccirilli, Angela Chiereghin, Brunella Guerra, Maria Paola Landini, Maria Grazia Capretti, Marcello Lanari, Tiziana Lazzarotto

**Affiliations:** 1 Operative Unit of Clinical Microbiology, St. Orsola-Malpighi General Hospital, University of Bologna, Via Massarenti 9, 40138, Bologna, Italy; 2 Operative Unit of Pathology, St. Maria Nuova Hospital, Reggio Emilia, Italy; 3 Operative Unit of Pathology, St. Orsola-Malpighi General Hospital, University of Bologna, Bologna, Italy; 4 Operative Unit of Obstetrics and Prenatal Medicine, St. Orsola-Malpighi General Hospital, University of Bologna, Bologna, Italy; 5 Operative Unit of Neonatology, St. Orsola-Malpighi General Hospital, University of Bologna, Bologna, Italy

**Keywords:** Cytomegalovirus, Congenital infection, Sensorineural hearing loss, Inner ear, Cochlea, Brain damage

## Abstract

**Background:**

Congenital cytomegalovirus (CMV) infection is a leading cause of sensorineural hearing loss (SNHL). The mechanisms of pathogenesis of CMV-related SNHL are still unclear. The aim is to study congenital CMV-related damage in the fetal inner ear, in order to better understand the underlying pathophysiology behind CMV-SNHL.

**Results:**

We studied inner ears and brains of 20 human fetuses, all at 21 week gestational age, with a high viral load in the amniotic fluid, with and without ultrasound (US) brain abnormalities. We evaluated histological brain damage, inner ear infection, local inflammatory response and tissue viral load.

Immunohistochemistry revealed that CMV was positive in 14/20 brains (70%) and in the inner ears of 9/20 fetuses (45%). In the cases with inner ear infection, the marginal cell layer of the stria vascularis was always infected, followed by infection in the Reissner’s membrane. The highest tissue viral load was observed in the inner ear with infected Organ of Corti. Vestibular labyrinth showed CMV infection of sensory cells in the utricle and in the crista ampullaris.

US cerebral anomalies were detected in 6 cases, and in all those cases, the inner ear was always involved. In the other 14 cases with normal brain scan, histological brain damage was present in 8 fetuses and 3 of them presented inner ear infection.

**Conclusions:**

CMV-infection of the marginal cell layer of the stria vascularis may alter potassium and ion circulation, dissipating the endocochlear potential with consequent SNHL. Although abnormal cerebral US is highly predictive of brain and inner ear damage, normal US findings cannot exclude them either.

## Background

Congenital cytomegalovirus (CMV) infection is a leading cause of sensorineural hearing loss (SNHL), occurring in 30-65% of children symptomatic at birth and in 7-15% of children with asymptomatic infection [1,2]. SNHL may be present at birth or with a late onset, after months or even years [3,4]. It can be unilateral or bilateral, with a wide range of severity and progression [5,6]. Approximately half of hearing losses due to congenital CMV infection are late-onset or progressive [7].

The mechanisms of pathogenesis of CMV-related SNHL are still unclear. Animal models have been studied to find CMV target cells in the inner ear and the role of associated inflammatory infiltrate [8-10]. The paucity of temporal bone autopsy specimens from infants with congenital CMV infection has hindered the critical correlation of histopathology with pathogenesis. Histopathology of SNHL was previously examined in a small number of human inner ears of fetuses at different gestational ages with abnormal brain ultrasound (US) findings [11]. The main finding was that CMV infection of the structures involved in endolymph production may cause potassium imbalance and subsequent degeneration of the sensory structures. However, further studies are necessary to explain the multiple factors involved in CMV-related SNHL.

We studied a wide autopsy series of fetal human inner ears all at 21 weeks of gestation, with and without US brain abnormalities and with a high viral load in the amniotic fluid. We examined histological brain damage, inner ear infection, local inflammatory response, and tissue viral load. The aim of our study is to further develop information about congenital CMV-related damage in the inner ear, especially cochlear infection, in order to better understand the underlying pathophysiology behind CMV-SNHL.

## Methods

### Subjects

Both inner ears and brains of 20 fetuses with congenital CMV infection documented at 21 weeks gestation were submitted for histological examination.

All the fetuses were from pregnant women with primary CMV infection arising before the 12^th^ week of gestation. Women who had anti-CMV IgM and anti-CMV IgG of low avidity or who seroconverted to CMV IgG positivity were classified as having primary infection. Fetal diagnosis of CMV infection was based on CMV positivity in amniotic fluid by culture and by Real Time polymerase chain reaction (PCR) at 21 weeks gestation. The viral load was more than 10^5^ copies/ml in all amniotic fluids.

At the time of amniocentesis, all pregnant women underwent US examinations that included a survey of all fetal organs [12]. Given the particular neurotropism of CMV, the intracranial anatomy was evaluated by targeted transvaginal neurosonographic examination, when permitted by the low position of the fetal head [13].

We studied 6 fetuses at 21 weeks gestation from CMV-seronegative women as negative controls. One case was a spontaneous miscarriage due to cervical incompetence and the other cases were elective terminations of pregnancy due to cardiac malformations (3 cases) and Spina bifida (2 cases).

The fetal tissues were analysed after obtaining the informed consent of the parents and in accordance with the policies of the Ethical Committee of St. Orsola-Malpighi General Hospital, Bologna, Italy and regulations of the Italian Ministry of Health, Rome, Italy.

### Histological examinations

The brain and both fetal inner ears were submitted for histological and immunohistochemical studies. Fetal inner ears were carefully removed from the temporal bone, left in a weak decalcifier (fixative Gooding Stewart, containing formaldehyde, Bio-Optica, Milano, Italy) for 24–36 hours, and then tangentially sectioned along the insertion of the VIII nerve.

Immunohistochemical staining for CMV early (ppUL44) antigen was performed to identify CMV-positive cells.

Brain damage was classified as severe, moderate and mild according to a score which considered and summarized each single lesion and the severity of the inflammatory infiltrate [14,15]. Severe damage included polymicrogyria, cortical necrosis, periventricular leukomalacia and microglial nodules. Moderate damage included telencephalic leukencephalopathy, microglial nodules and focal necrosis. Mild damage included telencephalic leukencephalopathy and rare microglial nodules.

The inflammatory infiltrate (B and T lymphocytes) was studied by immunohistochemical staining for CD3, CD4, CD8, CD20. Granzyme B expression was investigated as a marker of cytotoxic activation of immune effector cells.

The inflammatory infiltrate was scored semi-quantitatively to assess the density of CD8 T-lymphocytes in the different ear structures, similar to what was used in the brain [14]. The quantity was defined as: score 0: no lymphocytes detected; score 1: scanty; score 2: numerous; score 3: abundant. Another score was added according to the distribution of lymphocytes, such as: score 1: scattered lymphocytes; score 2: scattered lymphocytes and clusters. As a marker of activation, granzyme B was graded as a percentage of the immune effector cells observed: score 0: no activated cells; score 1: 1–10%; score 2: 11–25%; score 3; 26–50%; and score 4: > = 51%.

As a whole, the inflammatory response was evaluated by adding the three scores to each inner ear structure examined. The final score ranged from 0 to 9. A mild inflammatory response was between 1 and 3, a moderate response between 4 and 6, and a severe response between 7 and 9.

The following antibodies were used: anti-CMV (DDG9/CCH2, monoclonal mouse, diluition 1:80) obtained from DAKO (Glostrup, Denmark); anti-CD3 (2GV6, monoclonal rabbit, prediluited), anti-CD4 (SP35, monoclonal rabbit, prediluited), anti CD-8 (SP57, monoclonal rabbit, prediluited), anti-CD20 (L26, monoclonal rabbit, prediluited), anti-granzyme B (GrB7, polyclonal, prediluited) from Roche Diagnostics (Tucson, Arizona, USA).

### Virological examinations

The shell vial procedure was used for CMV isolation from amniotic fluid as described elsewhere [16]. DNA was extracted from 1 ml of amniotic fluid using the NucliSens easyMAG System (bioMerieux, Marcy l’Etoile, France), eluted in 25 μl of Elution Buffer and 20 μl were processed for CMV DNA quantification. DNA extraction from paraffin-embedded tissue was performed on 10 micron tissue slices using BioSprint 15 DNA Blood Kit (Qiagen GmbH, Hilden, Germany). Purified DNA was eluted in 80 μl of distilled water and quantified using Real-Time PCR assays (Quantifiler Human DNA Quantification Kit, Applied Biosystems, Foster City, California, USA). Five ng of DNA were processed for CMV DNA quantification.

CMV DNA was quantified using a Real-Time PCR assay (CMV ELITe MGB™ kit, ELITech Group, Trezzano, Milano, Italy). Amplification, detection and analysis were performed using the ABI PRISM 7300 platform (Applied Biosystems, Foster City, California, United States). The limit of detection for amniotic fluid was 16 copies/ml and viral load was reported as number of copies/ml. The limit of detection for tissue samples was 13 copies/5 ng of DNA and viral load was reported as number of copies/5 ng of DNA.

## Results

Immunohistochemistry for CMV early antigen performed on brains of 20 congenitally infected fetuses revealed that CMV was positive in 14 brains (70%) (Table 1).

**Table 1:** Virological, ultrasound and histological findings in 20 fetuses studied

**Case n°**	**Ultrasound findings**	**CMV immunohistochemistry**		**Histological brain damage**
		Brain	Inner ear	
1	Cerebral periventricular hyperechogenicity	P	P	severe
2	Cerebral periventricular hyperechogenicity, growth retardation, hyperechogenic bowel	P	P	severe
3	Cerebral ventriculomegaly, cerebral periventricular hyperechogenicity, hyperechogenic bowel	P	P	severe
4	Cerebral periventricular hyperechogenicity	P	P	moderate
5	Cerebral periventricular hyperechogenicity	P	P	moderate
6	Cerebral periventricular hyperechogenicity, hyperechogenic bowel	P	P	moderate
7	Normal	P	P	moderate
8	Normal	P	P	moderate
9	Normal	P	P	moderate
10	Normal	P	N	severe
11	Normal	P	N	moderate
12	Normal	P	N	moderate
13	Normal	P	N	moderate
14	Normal	P	N	mild
15	Normal	N	N	not present
16	Normal	N	N	not present
17	Normal	N	N	not present
18	Normal	N	N	not present
19	Normal	N	N	not present
20	Normal	N	N	not present

**P** positive; **N** negative.

Among 14 CMV-positive brains, 4 cases showed severe histological damage (29%), 9 cases showed moderate damage (64%) and 1 case showed mild damage (7%) (Table 1).

We found no evidence of brain CMV infection by immunohistochemistry in 6 out of the 20 fetuses studied (30%). No histological damage was observed in these 6 fetuses lacking evidence of brain infection (Table 1).

In all the 20 fetuses studied, CMV-positive cells were detected in all placentas and in at least one organ including pancreas, lungs, kidneys and liver; thus confirming congenital CMV infection.

At 21 weeks gestation, abnormal cerebral US findings were observed in 6 out of 20 fetuses (30%). At histology, brain damage was present in all of them; severe damage in 3 fetuses and moderate damage in 3 (Table 1).

Among the 14 fetuses with normal US in the brain and in the other organs, in 8 cases the brain was CMV-positive (57%) with a brain damage that was severe in 1 case, moderate in 6 and mild in one. The remaining 6 fetuses with normal US showed CMV-negative brain and no histological damage (43%) (Table 1).

Immunohistochemistry for CMV early antigen performed on both inner ears of the 20 fetuses revealed that CMV was positive in 9 fetuses (45%) (Table 1).

The inner ears were positive only in fetuses with CMV-positive brains.

Among the 9 fetuses with CMV-positive inner ears, 6 showed bilateral CMV-positivity. In 2 fetuses, only one inner ear was infected. In another fetus, only the left inner ear was available (Table 2). Therefore, the total number of CMV-positive inner ears was 15. Histological examination revealed that many structures were involved (Table 2).

**Table 2:** CMV-antigen and CD8 T-lymphocytes in the cochlea and in the vestibular apparatus in 9 fetuses with CMV-positive inner ear

**Case n°**		**Cochlea**					**Vestibular apparatus**				
		**Organ of Corti**	**Stria vascularis**	**Reissner’s membrane**	**Spiral ganglion**	**Cochlear nerve**	**Crista ampullaris**	**Semicircular canals**	**Saccule**	**Utricule**	**Vestibular ganglion**
1	R	NA	NA	NA	NA	NA	NA	NA	NA	NA	NA
	L	N/+	P/++	N/+	N/++	N/+	N/+	N/+	N/+	P/+	N/++
2	R	N/+	P/++	N/+	P/+	N/+	N/+	N/+	N/+	P/++	N/+
	L	P/+	P/+++	P/++	P/+	N/+	N/+	N/+	N/+	N/+	N/+
3	R	N/-	P/+	N/-	N/-	N/-	N/-	N/-	N/-	N/-	N/-
	L	N/-	P/+	P/-	N/+	N/-	P/+	P/+	N/-	P/+	N/-
4	R	N/+	P/++	N/++	N/+	N/+	N/+	N/-	N/+	P/+	N/+
	L	N/-	N/+	N/-	N/-	N/-	N/-	N/-	N/-	N/-	N/+
5	R	N/+	P/+++	P/++	N/+	N/+	P/+	N/+	N/++	N/++	P/+
	L	N/+	P/+++	P/+	N/+	N/+	P/++	N/+	N/++	N/++	P/+
6	R	N/+	P/+	P/+	N/+	N/+	N/-	N/-	N/-	N/-	N/-
	L	N/-	P/+	P/+	N/-	N/-	N/-	N/-	N/-	P/+	N/-
7	R	N/-	P/+	N/-	N/+	N/+	N/+	N/-	N/+	N/+	N/+
	L	N/+	P/++	N/+	N/+	N/+	N/-	N/-	N/-	N/-	N/+
8	R	N/-	P/+	N/-	N/+	N/+	N/+	N/-	N/+	N/+	N/+
	L	N/-	N/+	N/-	N/-	N/-	N/-	N/-	N/-	N/-	N/+
9	R	N/-	P/++	N/+	N/+	N/+	N/+	N/-	P/++	N/-	N/+
	L	N/+	P/++	P/+	N/+	N/+	N/+	N/-	P/+	N/+	N/+

**NA** not available; **R** right; **L** left; **P** CMV-positive; **N** CMV-negative; **-** no CD8 T-lymphocytes; **+** scanty CD8 T-lymphocytes; **++** numerous CD8 T-lymphocytes; **+++** abundant CD8 T-lymphocytes.

**Figure 1:**
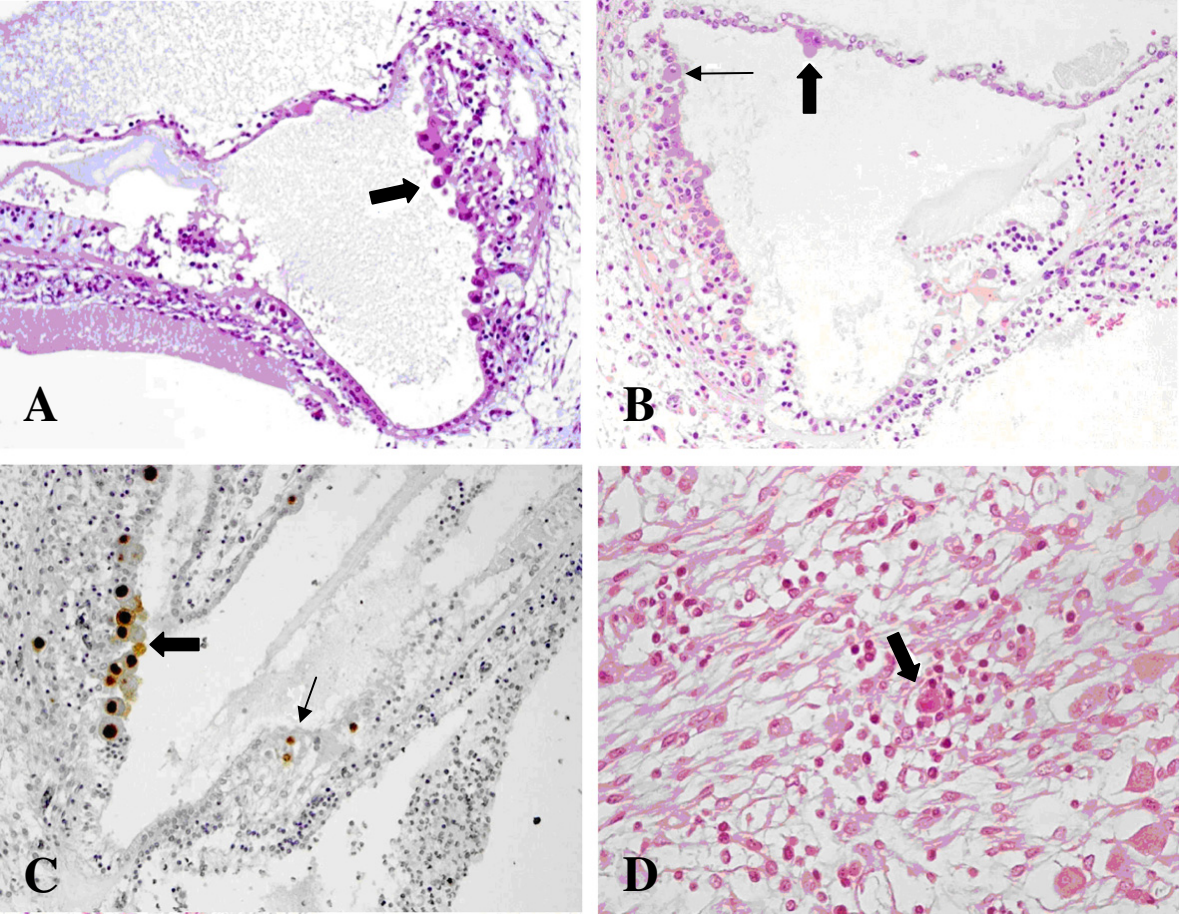
**CMV infection in the cochlea. A)** Numerous cytomegalic inclusions in the marginal layer of the stria vascularis (arrow). Haematoxylin and eosin (HE). **B)** Cytomegalic inclusions in the marginal layer of the stria vascularis (small arrow) and in the Reissner’s membrane (big arrow). HE. **C)** CMV immunohistochemistry showing strong nuclear CMV positivity in the marginal cell layer (big arrow) and in the Organ of Corti (small arrow). In the Organ of Corti, CMV-positive cells are most likely one ciliated cell (top) and one supporting cell (bottom). The other CMV-positive cell on the right is probably a supporting cell. **D)** Spiral ganglion: a cytomegalic neuron surrounded by lymphocytes. HE.

Regarding the cochlea, the superficial marginal layer of the stria vascularis was always CMV-positive (15 inners ears) (Figure 1A). The Reissner’s membrane was often infected (7 inner ears) (Figure 1B), whereas the Organ of Corti was found to be CMV-positive only in one fetus, in the left side (Figure 1C). In the same fetus, the spiral ganglion was infected, bilaterally (Figure 1D).

**Figure 2:**
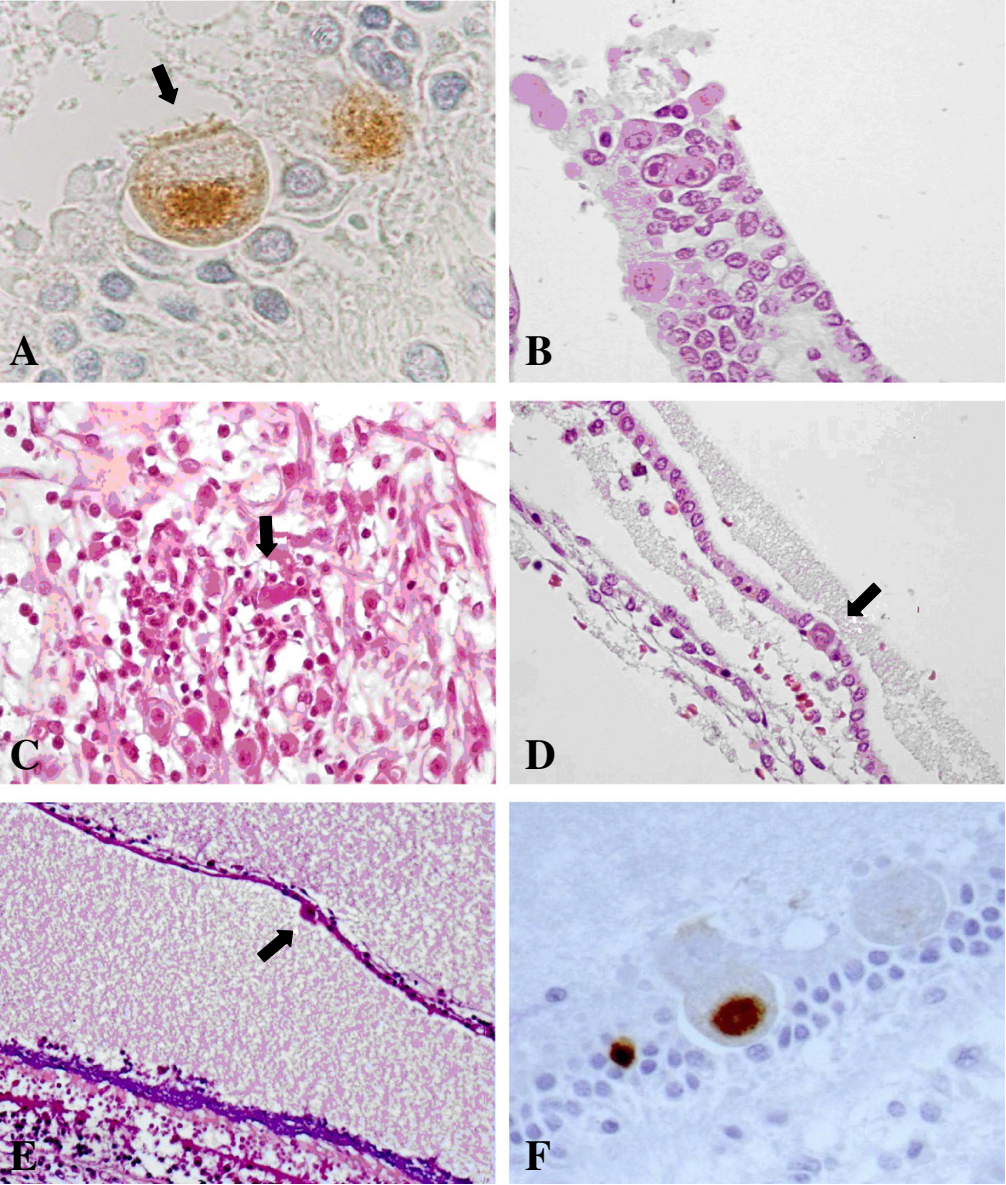
**CMV infection in the vestibular apparatus. A)** CMV immunohistochemistry showing CMV-positive cells within the utricle. The cell indicated by the arrow may likely be a sensory cell as suggested by the presence of cilia. **B)** Crista ampullaris: cytomegalic cells in the superficial epithelial layer. HE. **C)** Vestibular ganglion: a cluster of lymphocytes surrounding infected neurons (arrow). HE. **D)** One cytomegalic cell (arrow) in the epithelial layer of a semicircular canal. HE. **E)** Saccule: cytomegalic inclusion (arrow) in the membranous layer. On the bottom, there is the sensory macula with the otolith layer, strongly basophilic, and the hair cells underneath. HE. **F)** Saccule: cytomegalic cells within the macula. The positive cells may be a supporting cell (left) and a sensory cell (right). CMV immunohistochemistry.

Regarding the vestibular apparatus, utricule hair cells were found positive unilaterally in five fetuses (Figure 2A). In two fetuses, CMV-positive cells were observed in the sensory cells of the ampullary crest (Figure 2B). In one of them, the vestibular ganglion was infected (Figure 2C). In the other fetus, together with the ampullary crest, the contiguous semicircular canal showed CMV-positive cells (Figure 2D).

In one fetus, CMV-positive cells were observed bilaterally in the saccule, in the membranous layer and in the sensory cells of the macula (Figure 2E, F).

In control fetuses, brain and inner ears were always CMV-negative by immunohistochemistry.

**Figure 3:**
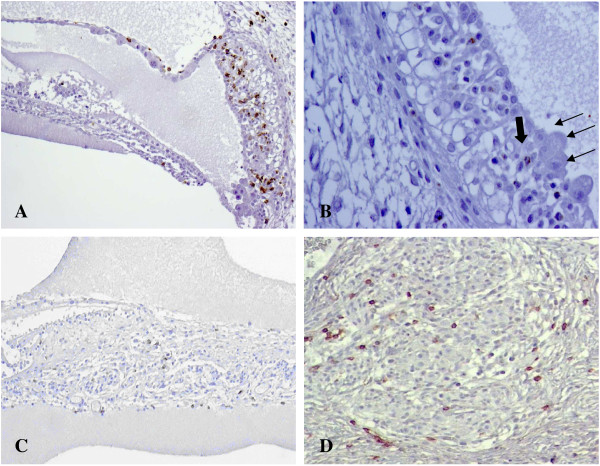
**Fetal inflammatory response in CMV-positive inner ears. A)** CD8 immunohistochemistry showing numerous CD8-positive lymphocytes in the epithelial layers of the stria vascularis and within the Reissner’s membrane. Lymphocytic infiltrate was mainly found in proximity to cytomegalic cells. **B)** Granzyme B immunohistochemistry in the stria vascularis: most of the lymphocytes close to CMV infected cells (small arrows) were activated expressing Granzyme B (big arrow). **C)** CD8 immunohistochemistry showing CD8-positive lymphocytes along the cochlear nerve fibers. **D)** CD8 immunohistochemistry showing CD8-positive lymphocytes within the spiral ganglion.

The inflammatory infiltrate, mainly composed of activated CD8 T-lymphocytes, Granzyme B-positive, was mostly found close to CMV-positive cells (Figure 3A, B). CD-8 T-lymphocytes, especially in clusters, were more abundant in the inner ear structures where the virus was detected (Table 2). No CD4 T-lymphocytes and CD20 B-lymphocytes were observed.

CD8 T-lymphocytes were also found along cochlear nerve fibers and in the spiral ganglion (Figure 3C, D). In the cochlear nerve, with no CMV-positive cells, the lymphocytes were not activated. In the spiral ganglion, CD8 T-lymphocytes, Granzyme B-positive, were always observed adjacent and near cytomegalic cells. There were also non-activated lymphocytes, more scattered and less associated with infected cells.

**Figure 4:**
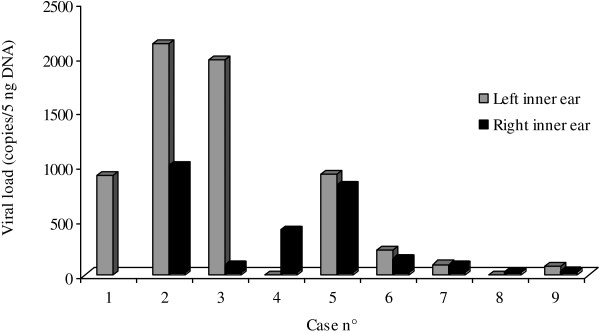
Tissue viral load in the 15 CMV-positive inner ears.

In the 15 CMV-positive inner ears, Real Time PCR detected a viral load between 13 and 2125 copies/5 ng DNA. The highest was observed in the inner ear with infected Organ of Corti (Figure 4). Among the CMV-negative inner ears and negative controls, Real Time PCR did not detect viral DNA.

## Discussion

**Figure 5:**
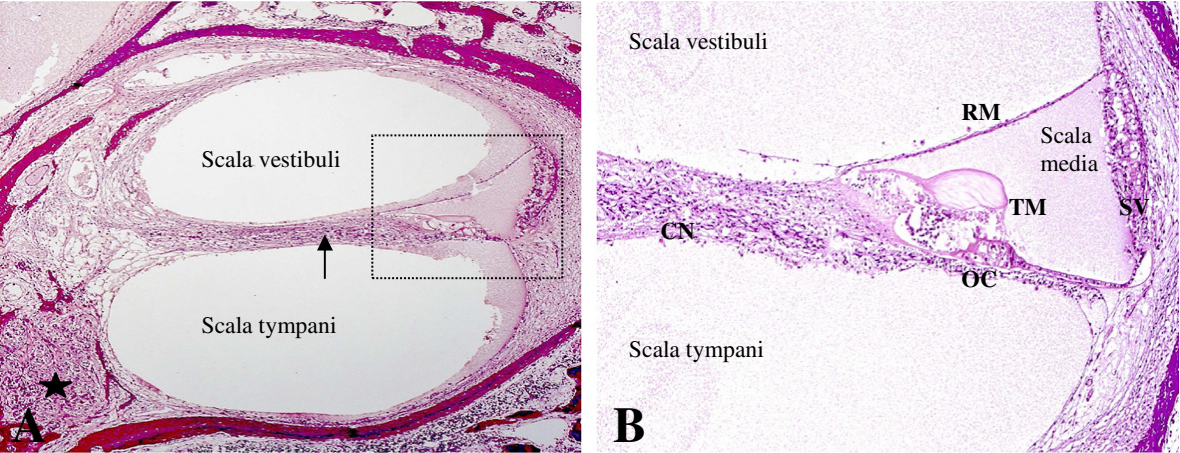
**Human fetal cochlea at 21 weeks gestation. A)** Cochlear turn: the cochlea is divided in three tubular compartments: scala vestibuli, tympani and media (detail). The first two contain perilymph, a liquid with an ionic composition similar to extracellular fluids. The scala media contains endolymph, with a positive potential of 80 mV, the endocochlear potential, essential for stimulating the sensory cells of the Organ of Corti. The signals generated travel along the cochlear nerve fibers (arrow) to the spiral ganglion (star) and then through the auditory pathway. **B)** Scala media: the scala media contains the Organ of Corti (OC), composed of sensory cells innervated by the cochlear nerve (CN) and stimulated by the tectorial membrane (TM) according to endolymph waves. Endocochlear potential within the scala media is mainly maintained by the stria vascularis (SV) with the contribution of the Reissner’s membrane (RM).

The inner ear contains the organ of hearing, the cochlea and the organ of balance, the vestibular labyrinth (Figure 5). The inner ear functions are briefly reviewed below in order to understand potential CMV damage.

The cochlea is a snail-shaped structure divided in three tubular compartments: the scala media, the scala tympani and the scala vestibuli. The scala tympani and vestibuli contain perilymph whose ionic composition is high in sodium and low in potassium. The scala media contains endolymph, high in potassium and low in sodium and calcium, which has a positive potential of +80 mV, the endocochlear potential [17,18]. Endocochlear potential positivity is maintained by potassium recycling, through many ion channels mainly regulated by the stria vascularis, composed of three layers of epithelial cells: the marginal towards the scala media, the intermediate and the basal. Between the intermediate and marginal cells there is the intrastrial space isolated from perilymph and endolymph, containing a fluid low in potassium and with a positive potential of +90 mV. Isolation of intrastrial space maintains the endocochlear potential and allows potassium diffusion [17,19-23].

Potassium activates the hair cells of the organ of Corti involved in mechano-transduction of sound waves, which projects to the spiral ganglion and, through the VIII (cochlear) nerve, to the cochlear nuclei in the brainstem [24-28].

The vestibular apparatus, responsible for the detection and maintenance of the equilibrium, is composed of the utricle, the saccule and the three semicircular canals. Signal mechano-transduction is regulated by the hair cells, located in the macula (utricle and saccule) and in the ampulla (semicircular canals). In both hair cell cilia are embedded in a gelatinous material, the otolithic membrane and the cupula, whose movements open or close different ion channels [29-31]. Dark cells are secretory cells located in the utricle and at the base of ampullae, which are absent in the saccule. They function in a similar way to the marginal cells of the stria vascularis, regulating potassium circulation and the endovestibular potential, which is +/−1 mV [32]. Hair cells take synapse with the vestibular nerve fibers that project to the vestibular ganglion, the first step of the vestibular pathway [33,34].

### CMV- related damage

As summarized and simplified above, the inner ear is a highly complex structure with delicate mechanisms and integrated neural connections. This being said, the functionality of the inner ear may be altered by CMV infection at many different levels with superimposed effects. Although the final result is mainly NSHL, little is known about cell-infection and damage with consequent altered physiology. Analysing the type of cell and the structures infected by CMV, the most likely pathophysiological events may be at least speculated.

### The cochlea

Considering our immunohistochemical results, the stria vascularis was found to be always CMV-positive in the marginal cell layer. As already mentioned, these cells are fundamental in keeping the endocochlear potential positive through potassium recycling. CMV-infection may alter the delicate ion circulation throughout the stria vascularis, damaging the ion channels and dissipating the positive potential of the intrastrial space. Absent or altered potassium channels, as reported in animal models or in genetic diseases, are responsible of hearing loss [23,35]. However, whatever affects endocochlear potential formation may potentially result in SNHL. For example, abnormal homeostasis of other ions, chloride and sodium, or gap junctions damage [36,37]. Without the endocochlear potential, the hair cells of the Organ of Corti may degenerate due to excessive calcium concentration in the endolymph or they may not simply be activated due to the lack of further signal transmission [32].

CMV-positive cells were also often observed in the Reissner’s membrane. This may result in electro-chemical imbalance between endolymph and perilymph. CMV-infection may also damage sodium and chloride channels, which contribute to endocochlear potential.

CMV-positive cells in the Organ of Corti were detected only in one fetus (case number 2), unilaterally. Two were supporting cells, though one is a ciliated cell corresponding to an inner hair cell (Figure 1C). Supporting cells play a role in survival of hair cells and spiral ganglion neurons. While in some species hair cell may regenerate, this event does not happen in mammals [38-40].

CMV infection in the hair cells may impair their functionality, altering the kinetic of stereocilia, cytoskeleton and in the outer hair cell, prestin contractility [41-43].

Regarding the aforementioned case with CMV-positive Organ of Corti, positive-cells were also observed in the spiral ganglion, bilaterally. Also neurons of the spiral ganglion cannot be replaced.

Moreover, in this inner ear the highest CMV-load was detected. Therefore, these findings suggest that this fetus, at birth, may have been hearing impaired.

Severity of inflammatory infiltrate, T-CD8 activated lymphocytes, must be taken into account as a contributory factor to hair cell damage, either directly, destroying infected cells, or indirectly, through cytokine secretion. Inflammatory mediators, as seen in prolonged infection, may also affect the other components of the scala media, such as the spiral ligament, inducing fibrosis [8-10]. Lymphocytes within the cochlear nerve, and spiral ganglia may also affect nerve conduction and integrity of the auditory pathway.

Non-activated CD8 T-lymphocytes were present along the cochlear nerve fibers and in the spiral ganglion, not in association with CMV-positive cells. Their presence probably reflects an extensive and ongoing inflammatory process with lymphocyte recruiting.

### The vestibular labyrinth

In our group of fetuses, the vestibular apparatus showed less structures infected by CMV compared to the cochlea. The utricule, with infected hair cells of the macula, was the most involved with five cases. In two cases, the sensory cells of the crista ampullaris showed CMV-infection. The saccule was bilaterally found only in one case, involving the sensory hair cells and the epithelial cells of the membranous layer. The semicircular canals were the least infected with only one case, unilaterally. These findings differ from what has been observed in the past, in which sensory cells of the macula and the crista ampullaris were always spared, instead affecting supporting cells and dark cells [11,44]. As already mentioned, dark cells play a part in the formation of the endovestibular potential, regulating potassium secretion, and their damage may further contribute to SNHL [32].

Furthermore, vestibular CMV infection may be a rare cause of delayed endolymphatic hydrops, which consists of development of hydropic otologic symptoms in the setting of prolonged deafness [45].

Although these findings suggest that CMV may be involved in vestibular functionality, newborns with congenital CMV infection are not routinely tested since CMV is not considered to play a significant role in vestibular pathway alteration. It should be noted however that some authors have reported abnormal vestibular tests in congenital infected babies [44,46,47].

### Inner ear and brain damage

US brain abnormalities at 21 weeks gestation were detected in six fetuses (30%), and in all these cases the brain revealed a histological damage and the inner ear was CMV-positive. This confirms that the inner ear is often involved in severely infected babies.

US has a sensibility in predicting histological brain damage, in case of high viral load in amniotic fluid, of 43% (6/14) and in predicting CMV-positive in inner ears of 67% (6/9).

In the cases of normal US findings, we observed a histological brain damage in eight fetuses (57%) and a CMV-positive inner ear in three fetuses (21%). Therefore a normal US cannot exclude brain or inner ear infection.

## Conclusion

The present study investigated the brain and the inner ear of 20 fetuses from women primarily infected by CMV before 12 weeks gestation and with a positive prenatal diagnosis. This study confirms the findings of a previous paper [11] and support the pathophysiological mechanisms suggested. However, we examined a larger number of fetuses and our series had the same characteristics: maternal primary infection during the first week of pregnancy, high viral load in the amniotic fluid and histology studies performed in fetuses at the same gestational week (21 weeks).

In the cases with inner ear infection, the marginal cell layer of the stria vascularis was always infected, followed by infection in the Reissner’s membrane. This infection may affect endocochlear potential formation and ion homeostasis, especially potassium recycling.

Vestibular labyrinth also showed CMV infection in sensory cells in the utricle as well as in the crista ampullaris. The clinical consequence of these findings are still unknown even though some authors have reported the possibility of carrying out vestibular tests in congenital infected babies [44,46,47].

Moreover, CMV Real Time revealed a correlation between tissue viral load and the number of inner ear structures with tissue damage and inflammatory infiltrate.

Inner ear CMV infection was also correlated with cerebral US and histological findings. All the cases with abnormal brain US findings revealed histological inner ear infection. However, a normal brain scan alone cannot exclude brain and inner ear damage.

## Abbreviations

CMV: Cytomegalovirus; SNHL: Sensorineural hearing loss; US: Ultrasound; PCR: Polymerase chain reaction.

## Competing interests

The authors declare they have no competing interests.

## Authors’ contributions

LG, MPB and TL conceived of the study, participated in its coordination and drafted the manuscript. DS, GP, AC, BG, MPL MGC and ML undertook the acquisition of data. All authors read and approved the final manuscript.
